# Striving to Fill in Gaps between Clinical Practice and Standards: The Evolution of a Pan-Canadian Approach to Patient-Reported Outcomes Use

**DOI:** 10.3390/curroncol29050296

**Published:** 2022-05-19

**Authors:** Amanda Caissie, Robert Olson, Lisa Barbera, Jennifer O’Donnell, Carol-Anne Davis, Jennifer Croke, Louise Bird, John Kildea, Erika Brown, Michael Brundage, Michael Milosevic

**Affiliations:** 1 Department of Radiation Oncology, Dalhousie University, Halifax, NS B3H 1V7, Canada; carol-anne.davis@nshealth.ca; 2 Department of Radiation Oncology, University of British Columbia, Prince George, BC V2N 4Z9, Canada; rolson2@bccancer.bc.ca; 3 Department of Oncology, University of Calgary, Calgary, AB T2N 1N4, Canada; lisa.barbera@albertahealthservices.ca; 4 Department of Radiation Oncology, Queen’s University, Kingston, ON K7L 5P9, Canadamichael.brundage@krcc.on.ca (M.B.); 5 Radiation Medicine Program, Princess Margaret Cancer Centre, Department of Radiation Oncology, University of Toronto, Toronto, ON M6B 1V2, Canada; jennifer.croke@rmp.uhn.ca (J.C.); mike.milosevic@rmp.uhn.ca (M.M.); 6 Canadian Partnership for Quality Radiotherapy, Carlyle, SK S0C 0R0, Canada; loub1965@icloud.com; 7 Medical Physics Unit, McGill University, Montreal, QC H3A 2T5, Canada; john.kildea@mcgill.ca; 8 Canadian Organization of Medical Physicists, Ottawa, ON K2K 2E2, Canada; edgconsulting@gmail.com

**Keywords:** patient-reported outcomes, quality improvement, radiotherapy, Big Data

## Abstract

Despite the known importance and necessity of the standardized collection and use of patient-reported outcomes (PROs), there remain challenges to successful clinical implementation. Facilitated through a quality improvement initiative spearheaded by the Canadian Partnership for Quality Radiotherapy (CPQR), and now guided by the Canadian Association of Radiation Oncology (CARO)’s Quality and Standards Committee, patient representatives and early-adopter radiation treatment programs continue to champion the expansion of PROs initiatives across the country. The current review discusses the evolution of a pan-Canadian approach to PROs use, striving to fill in gaps between clinical practice and guideline recommendations through multi-centre and multidisciplinary collaboration.

## 1. Introduction

Patient-reported outcomes (PROs) have been shown to play a critical role in oncology survivorship [1] by facilitating symptom management during and after cancer treatment, improving patient–health-care-provider communication [2], quality of life [3] and survival [4]. PROs can be used to benefit individual patients through point of care clinical use, assessing the impact of cancer and treatments on their physical and psychosocial well-being. There is also the potential to positively impact the health care system as a whole through widespread PROs implementation and Big Data initiatives aiming to understand how specific treatments, such as radiotherapy, can affect patient outcomes, including PROs, alongside the more traditional outcome measures of tumour recurrence, mortality and treatment side effects.

## 2. Development of the Canadian Partnership for Quality Radiotherapy (CPQR)’s PROs Initiative

Under the guidance of the CPQR, a partnership of Canadian radiotherapy professional societies (the Canadian Association of Radiation Oncology—CARO, Canadian Organization of Medical Physicists—COMP and Canadian Association of Medical Radiation Technologists—CAMRT), pan-Canadian quality improvement (QI) initiatives, including those for PROs, have been feasible, with a relatively small number of cancer centres that provide radiotherapy services (46 total) across the country. Since CPQR’s beginning in 2010, its iterative approach to QI initiatives has supported its evolution to become a leader in Canadian radiotherapy QI and safety [5,6,7]. Since its release of “Quality Assurance Guidelines for Canadian Radiation Treatment Programs” in 2011 [8], CPQR has included reviews of patient outcomes, such as treatment-related toxicity, disease control and survival, amongst its key quality indicators of a radiotherapy program. Since the key quality indicators were embedded into the Accreditation Canada radiotherapy standards in 2017 [9], there is now an expectation that programs not only review physician-reported disease control and toxicity outcomes but also PROs. The Accreditation Canada standards focused attention clearly on the patient with the term “client reported outcomes”. In 2017, CPQR launched a pan-Canadian PROs initiative to support centers striving to meet the accreditation requirement of PROs program implementation.

Standardized use of PROs has been supported with the aim to not only improve the clinical encounter for individual patients but also facilitate Big Data efforts that will enable “real world” data sharing and analysis. With the ultimate goal of facilitating population-based understanding of treatment outcomes, including PROs, there is a recognized need to standardize how and what data are collected. The decision to include PROs and Big Data in CPQR’s mandate came following a CPQR pan-Canadian survey of compliance identifying the two key quality indicators related to patient outcome reporting to be the most challenging to achieve for programs. The priority of PRO and Big Data also came through in a survey of radiation thought leaders across Canada who were asked to identify future patterns and trends that would most likely influence quality of care and the evolution of our field over the next 5 to 10 years. CPQR, therefore, aimed to create a guidance document [10] that could foster a shared understanding of the challenges associated with local PRO use, together with a reasonable expectation of the infrastructure required for successful implementation. The objective was to promote the consistent and routine collection of PROs through nationally endorsed, validated PRO measures [11].

## 3. CPQR’s PRO Guidance Developed

In 2018, a review was written to outline CPQR’s planned multipronged approach, beginning with a convened working group (Olson, R., Barbera, L., Caissie, A., Davis, C.-A., Milosevic, M., Brown, E.) to support harmonization of PRO use and assessment nationally [7]. With a representative from each Canadian radiotherapy centre on CPQR’s National Quality Assurance Committee, CPQR was able to identify individuals considered to be the most knowledgeable in the use of PROs. From July to November 2018, semi-structured interviews were held with these individuals from 20 centres across Canada, representing Ontario, Quebec, the Atlantic provinces, Alberta, Manitoba and British Columbia. All the centres endorsed the use of PROs, although numerous barriers were identified, including, but not limited to, lack of resources (i.e., IT infrastructure or support for psychosocial needs), staff buy-in, knowledge of tool(s) to implement and provincial support as well as patient exhaustion with many questionnaires. The desire to learn from other Canadian centres was clearly expressed, as was the interest in guidance from CPQR in terms of what should be measured and how to measure it. Based on information gleaned through this environmental scan and a robust literature review, the PRO working group drafted a series of guidance statements designed to address identified gaps in the collection and use of PROs. The 11 guidance statements were then refined and validated through a Delphi-consensus process, resulting in the final “Guidance on the Use of Patient Reported Outcomes for Canadian Radiation Treatment Programs” [10].

The stepwise guidance helps radiotherapy programs identify target patient populations, establish benchmarks for the frequency of outcome collection and inform the development of process maps to support a program-level response to patient scores. With the recommendation that programs use CPQR-endorsed tools where relevant or appropriate, CPQR’s website [11] includes a shortlist of standardized PROs measures, including the general symptom screen tool of the Edmonton Symptom Assessment System-revised (ESAS-r) as well as disease-site-specific measures, prioritized based on literature review and an awareness of the high symptom burden in certain tumour sites (Table 1). In the absence of a nationally endorsed PRO measure for certain disease sites, it has been recommended that centres use a rigorous process for PRO measure selection. Much of CPQR’s work to formally evaluate disease-specific PRO measures was completed through collaboration with Cancer Care Ontario/Ontario Health, illustrating how CPQR has been successful by operating within an integrated, collaborative network. The importance of industry partners in this collaborative work should also be noted, identifying ways to integrate PROs measures and electronic PROs (ePROs) platforms within electronic medical records.

## 4. The Patient Voice in PROs

Strong patient engagement has been a key success for CPQR, with patients as active participants in the planning and implementation stages of all QI initiatives. From the very beginning, there has been patient involvement at the CPQR Steering Committee level, with a strong patient influence on the decisions to include patient outcomes as key quality indicators and develop the CPQR PROs guidance document. CPQR’s patient representatives have co-authored the various guidance documents, including that of PROs as well as the earlier “Patient Engagement Guidance for Canadian Radiation Treatment Programs” of 2016, which included a statement for PROs: “The radiation treatment program collects PRO and works to increase the number of patients from which these data are collected” [12]. As with all other CPQR initiatives, the PRO initiative benefited from concerted efforts for knowledge translation through various workshops and presentations at Canadian and international conferences. These conferences have proven instrumental as catalysts for culture change and to gain grassroots buy-in that the effort and resources required for QI initiatives, such as PRO, are well worth the investment for patient care. Presenting at many of these conferences, CPQR’s patient representatives have clearly articulated their passion for improving care by including the patient’s voice in every aspect of the care trajectory. To quote but a few of their powerful messages: “what do you mean you don’t know the outcomes of your treatments and how patients tolerate it? Everyone is unique and by using PROs, you the practitioner are expanding your knowledge base everyday to provide the best quality of life to your patients now and in the future”. To complement the physician assessment of clinical outcomes, PROs are designed to capture patients’ perspectives on care. Radiotherapy program implementation of PROs is, therefore, a means of promoting patient engagement in everyday clinical decisions.

## 5. Expansion of PROs across Canadian Radiotherapy Centres

At the onset of the PROs initiative in 2017, a few individual Canadian radiotherapy centres and research teams had made considerable advances to enable PROs data collection. From 2017 to 2019, CPQR supported the translation of lessons learned from PROs leads to other programs across the country. With geographically distributed pilot projects aimed to support PROs “early adopter” radiotherapy programs, CPQR hoped to capitalize on the existing PRO programs with the infrastructure and expertise that had been siloed to that point.

Ontario has had a long history of PROs use since creation of the Cancer Care Ontario/Ontario Health’s PROs and Symptom Management Program in 2007, implementing routine symptom screening at a system level in ambulatory cancer clinics [13]. The electronic Interactive Symptom Assessment and Collection (ISAAC) tool was created and used by the majority of regional cancer centres. It includes the ESAS-r and the patient-reported Eastern Cooperative Oncology Group performance status tool (pECOG). Symptom screening rates currently represent a quality indicator for programmatic performance in all regional cancer centers [14]. Provincial symptom screening in the real-world setting has been shown to decrease emergency room visits, hospital admissions and risk of suicide as well as improve access to psychosocial care and overall survival [15,16,17,18,19]. Work is ongoing to assess the benefits of provincially implemented disease-site-specific PROs, such as the EPIC-Clinical Practice [20,21], with studies of feasibility and acceptability showing positive feedback from patients and providers alike.

Within the context of a provincial PROs program, the radiation medicine program at Tom Baker Cancer Centre routinely uses the ESAS-r and Canadian Problem Checklist (CPC) at first and last radiotherapy review visits. Symptom scores and the clinical team’s response are documented by nursing in the electronic medical records using a template. The brachytherapy program routinely uses the American Urology Association Symptom Score Questionnaire for men with prostate cancer. Symptoms are documented at one to two time points after treatment to monitor and manage acute toxicity. The head and neck team has recently completed a pilot investigation of the MDASI-HN, with the expectation that the results will inform future decisions about routine implementation. The Patient Reported Measurement Program with Cancer Care Alberta is currently designing a revised approach to collecting general and disease-specific PROs from patients via their personal mobile devices, with the results feeding directly into the electronic medical record.

At the Cedars Cancer Centre of the McGill University Health Centre in Montreal, a patient portal mobile phone app is being used to collect ePROs, with commonly used PRO measures, such as ESAS-r and EPIC, as well as in-house questionnaires and satisfaction surveys. The design and development of the ePRO tool was conducted in-house through a process of participatory stakeholder co-design led by a radiation oncologist, a medical physicist and a patient who was also a computer scientist [22]. Using the patient-facing mobile phone app, patients access their medical data entered into the oncology information system by their clinical team, and, using the clinician-facing dashboard, clinicians access ePROs data entered by patients. Both patients and their clinical teams are informed and empowered by the two-way information exchange.

In British Columbia, collection and utilization of PROs for routine clinical care was developed through the Prospective Outcomes and Support Initiative (POSI) [23,24,25,26,27,28]. Since its inception in 2013, POSI has increased its PROs collection uptake to approximately 25,000 PROs per year across all six cancer centres. In addition to routine use in clinical care, the PROs collected have guided research in bone metastases [27,28], head and neck, breast, gynecological and lung cancer patients. Plans are underway to combine data with the other Canadian programs collaborating under CPQR’s early adopter initiative.

Recognizing the power of communities of practice to help collaborating members overcome barriers and avoid duplication of efforts/resources [29], CPQR’s “early adopters” included programs leading in PROs alongside Dalhousie University’s Department of Radiation Oncology (DRO), which had a willingness to change the culture and implement standardization but lacked the critical electronic infrastructure to make meaningful change. Dalhousie University’s DRO is composed of four centres (Halifax, Sydney, Saint John, Charlottetown) across three provinces. While several DRO centres have a longstanding history of PROs use, their programs have been paper-based since a 2009 Canadian Partnership Against Cancer grant enabled two centres to begin PROs screening using the CPC and ESAS-r. Appreciating the drawbacks of paper-based screening that affect the efficiency and efficacy of clinical programs and limit research opportunities, three Dalhousie centres were awarded grant funding in 2019 to support ePROs. A department–wide ePROs program was launched in September 2021, with a phased approach across centres and tumour sites so that user feedback can inform the roll-out. Standardized clinic workflows now include ePROs at CT simulation, first and last radiotherapy review. The ESAS-r and CPC are used, with the roll-out of other CPQR-endorsed questionnaires planned following the BPI, which was the first tumour-site-specific PRO measure implemented. Research unit support was invaluable to navigate IT project complexities, including vendor/collaborator contracts, privacy impact assessments and IT architectural reviews for the ePROs application. While multi-centre implementation of an ePROs program has been feasible with multi-stakeholder collaboration, the process was complex and time-intensive. It was the hope of CPQR’s initial pilot project that other centres aiming to transition from paper-based to ePROs systems may benefit from the many lessons learned through an IT-dependent QI project of this scope.

## 6. PROs Collection and Use in the Era of COVID-19

**Figure 1:**
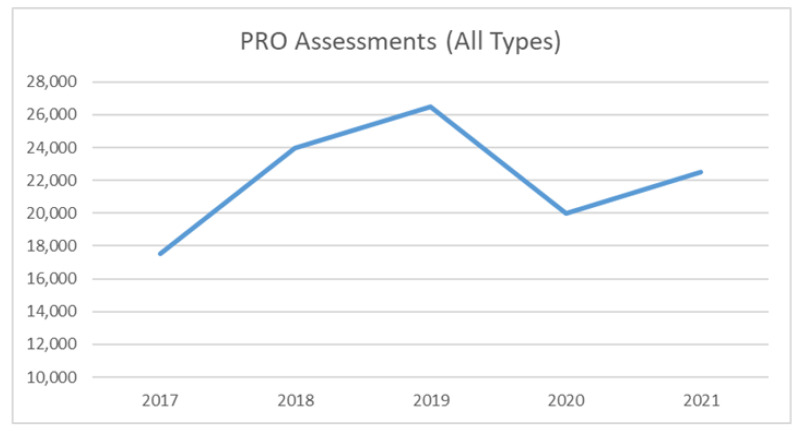
Number of PROs assessments captured in British Columbia’s POSI.

The COVID-19 pandemic and its resultant restrictions within the health care system have affected the pan-Canadian PROs initiative in both negative and positive ways. As shown in Figure 1, there was a small decrease in British Columbia’s radiotherapy PROs collection at the beginning of the COVID-19 pandemic. There has been a subsequent rebound, although PROs collection has not yet returned to pre-pandemic levels. PROs data collection in Alberta was affected similarly by the pandemic. The shift to virtual care resulted in a dramatic decrease in PROs completion, which is still recovering.

Upon declaration of the COVID-19 pandemic in March 2020, Ontario PROs collection ceased completely due to the potential infection risk of completing PROs on shared devices (e.g., kiosks and iPads) and the rapid transition to virtual care, with mechanisms of PROs collection at home not established. In September 2020, the Princess Margaret Cancer Centre developed and implemented “PM at Home” (PaTH), a remotely accessible/web-based ePROs platform. The current workflow for PaTH includes: (i) the patient receives an email two days prior to the clinical encounter with a link to the PROs questionnaire, (ii) the patient completes the PROs questionnaire on their personal device, (iii) responses are available to health care providers and are integrated within the patient’s electronic medical record [30]. Management guides for each symptom within the ESAS-r and colour-coded schema for symptom severity (e.g., mild: green, moderate: yellow, severe: red) enable triaging of clinic patients and tailored interventions [31]. Future opportunities within PaTH include customization for disease-specific items and thresholds for intervention, as well as correlation of PROs with clinical and treatment characteristics, including radiotherapy dosimetry.

Although the coordination and timing of Dalhousie’s DRO large-scale PROs initiative were challenged by COVID-19 restrictions, the greatest bottleneck of project implementation (IT approvals) saw an escalation in priority during the pandemic, with ePROs recognized as a powerful tool to assess patient symptoms in a clinic or remotely. At the McGill University Health Centre, the patient portal mobile phone app was used to send patients COVID-19 screening questionnaires prior to arrival at the hospital, and the virtual check-in feature allowed patients to wait in their cars and complete their screening questionnaire there before being called in for their treatments.

## 7. The Future of the Pan-Canadian PROs Initiative

**Figure 2:**
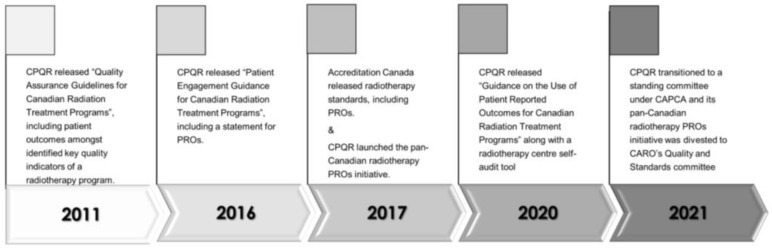
Timeline of key events in the evolution of CPQR’s pan-Canadian RT PRO initiative.

Initially supported through financial and strategic backing from the Canadian Partnership Against Cancer, in October 2021, CPQR transitioned to become a standing committee within the Canadian Association of Provincial Cancer Agencies (CAPCA). With a board that is comprised of the CEOs (or their delegates) of the 10 provincial cancer agencies, CAPCA and its new CPQR Quality Committee are well positioned to exert a top-down influence that complements the bottom-up approach that has been so successful for CPQR over the past 10 years. When attempting to create system-level change, there is a recognized need for the involvement of local champions. CPQR will maintain its model of a pan-Canadian radiotherapy network hub inclusive of representatives from each of the 46 centers, promoting QI at the grassroots level and providing CPQR with frontline feedback. With PROs being but one of the many key quality indicators for radiotherapy, sustained support of a dedicated pan-Canadian PROs initiative was sought as the CPQR framework changed. In the fall of 2021, the PRO initiative was divested to CARO’s Quality and Standards Committee, which now aims to follow the iterative process that has been key to the success of all the other CPQR’s initiatives to date (Figure 2).

While PRO guidance has been developed, challenges remain to bridge the gap between standards and clinical practice. In the preliminary phases of its work, CPQR’s PRO working group identified common challenges to PRO implementation: a need for “buy-in” from clinicians and health authorities to prioritize PROs alongside physician-rated toxicities, promotion of PRO use beyond research to the point of patient care as well as clinical action plans and sufficient resources to deal with patient issues identified. It is suspected that many radiotherapy centres still lack the resources for PRO collection and use, from human resource to electronic infrastructure, that is required to fill in the “survivorship gap” and enable remote PRO assessments outside of clinics. Knowledge translation efforts, including, but not limited to, the current review, aim to promote developed standards and endorsed questionnaires while raising awareness of PRO practices amongst “early adopter” centres (Table 2). In 2020, a self-audit tool was released, allowing programs to evaluate their alignment with PRO guidance [32]. There is now a need for a phase of community feedback in order for the pan-Canadian PRO initiative to understand the current PRO facilitators and barriers. CARO is also a member organization of the PROTEUS Consortium (Patient Reported Outcomes Tools—Engaging Users and Stakeholders, theproteusconsortium.org, accessed on 3 May 2022), which aims to address barriers to PRO implementation by disseminating established tools designed to guide users and stakeholders through the PRO implementation journey. Through continued collaboration with CPQR, it is hoped that the pan-Canadian PRO initiative will maintain its momentum under CARO, with a commitment to high-quality patient-centered care reflected in its new role as the steward of CPQR’s patient-centered programs.

The pan-Canadian PROs and Big Data initiatives aim to facilitate data analysis of treatment and patient outcomes that may be used to inform population-based decisions regarding optimal treatment delivery. While there is an important need for large-scale, multi-institutional, real-world data, Big Data efforts are currently in an early phase of development, limited by a lack of standardized data for collection and electronic infrastructure for data analysis that is not as resource-exhaustive as manual approaches. International society-led QI initiatives are currently underway to promote the standardization of data, with CPQR/CARO’s PROs initiative contributing alongside others, such as the American Association of Physicists in Medicine’s recently released draft of the Operational Ontology for Radiation Oncology, which includes PROs in its prioritized set of key data elements, attributes and relationships [33].

## 8. Conclusions

As radiotherapy departments begin to adopt the same PROs measures and consistent collection time points, time will tell whether efforts to date have paved the way for future Big Data analysis that can evaluate patient-, diagnosis- and treatment-related factors affecting oncology patient outcomes, including PROs.

## Figures and Tables

**Table 1:** CPQR’s endorsed PRO measures.

Disease Site	PRO Measure
All cancer (general)	Edmonton Symptom Assessment Screening (ESAS)
Bone metastasis	Brief Pain Inventory (BPI)
Prostate cancer	Expanded Prostate Cancer Index Composite (EPIC)
Head and neck cancer	MD Anderson Symptom Inventory for Head and Neck (MDASI-HN)
Gynecological cancer	Cervical cancer-specific quality of life module of the European Organization for Research and Treatment of Cancer (EORTC QLQ-CX24)

**Table 2:** List of co-authors’ (and CPQR PRO working group members’) publications contributing to the pan-Canadian RT PROs initiative since its launch in 2017.

Publication	Publication Year	Publication Party/Society	Purpose	Relevance to PRO Promotion
Caissie et al. [7]	2018	CPQR	Develop a pan-Canadian approach to PRO integration in radiation oncology	Promote a pan-Canadian approach to PRO use and the future goal of linking PRO data to RT plan parameters
Olson et al. [23]	2018	BCCA	Initiate prospective PRO collection to guide care, quality improvement and research	The Prospective Outcomes and Support Initiative (POSI) has facilitated PRO collection, leading to research opportunities and change in care
Brundage et al. [20]	2019	Multi-institutional-Ontario	Introduce a PRO (EPIC-CP) in routine ambulatory care of prostate cancer and assess acceptability by clinicians and patients	EPIC-CP was endorsed among clinicians and patients Provincial implementation was recommended
Barbera et al. [15]	2020	ICES	Examine the effect of generic PRO (ESAS) exposure on survival	ESAS exposure was associated with improved survival
Barbera et al. [18]	2020	ICES	Examine the effect of generic PRO (ESAS) exposure on emergency department visits and hospitalization	ESAS exposure was associated with decreased rates of emergency department visits and hospitalizations
Montgomery et al. [13]	2020	CCO	Recommend an approach for PRO selection, implementation and evaluation for use in a clinical setting using an example from head and neck cancer	PRO selection was assessed through performance in three overarching themes: symptom coverage, usability and psychometric properties PRO implementation was assessed through three concepts: acceptability, outcomes and sustainability
Atallah et al. [21]	2021	Multi-institutional-Ontario	Implement a cervix-cancer-specific PRO in gynecologic oncology clinics	Implementation of the PRO (EORTC QLQ-CX24) in gynecologic oncology clinics was feasible and acceptable by clinicians and patients
Sit et al. [27]	2021	BCCA	Investigate potential relationships between tumour biology/histology and PROs	Breast cancer subtype was found to have an impact on PRO/symptom improvement post-palliative RT for bone metastases
Olson et al. [28]	2022	BCCA	Assess advanced RT technique through PRO (BPI) in patients with bone metastasis	Advanced RT techniques were not associated with a significant difference in PRO (BPI) for patients with bone metastasis

BCCA, British Columbia Cancer Agency; BPI, Brief Pain Inventory; CCO, Cancer Care Ontario; CPQR, Canadian Partnership for Quality Radiotherapy; EPIC-CP, Expanded Prostate Cancer Index Composite for Clinical Practice; EORTC QLQ-CX24, European Organization for Research and Treatment of Cancer Quality of Life Cervical Cancer module; ESAS, Edmonton Symptom Assessment System; ICES, Institute for Clinical Evaluative Sciences; PRO, patient-reported outcomes; RT, radiotherapy.
